# Safety of heparin bridging therapy for transrectal ultrasound-guided prostate biopsy in patients requiring temporary discontinuation of antithrombotic agents

**DOI:** 10.1186/s40064-016-3610-6

**Published:** 2016-11-04

**Authors:** Itsuto Hamano, Shingo Hatakeyama, Tohru Yoneyama, Yuki Tobisawa, Osamu Soma, Teppei Matsumoto, Hayato Yamamoto, Atsushi Imai, Takahiro Yoneyama, Yasuhiro Hashimoto, Takuya Koie, Chikara Ohyama

**Affiliations:** 1 Department of Urology, Hirosaki University Graduate School of Medicine, 5 Zaifu-cho, Hirosaki, 036-8562 Japan; 2 Department of Advanced Transplant and Regenerative Medicine, Hirosaki University Graduate School of Medicine, Hirosaki, Japan

**Keywords:** Antithrombotic agents, Discontinuation, Heparin bridging, Prostate biopsy

## Abstract

**Background:**

Safety of heparin bridging therapy for transrectal ultrasound-guided prostate (TRUS) biopsy in patients requiring temporary discontinuation of antithrombotic therapy is unknown. This study aimed to assess the relationship between heparin bridging therapy and the incidence of complications after TRUS biopsy.

**Methods:**

From January 2005 to November 2015, we performed 1307 consecutive TRUS biopsies on 1134 patients in our hospital. The patients were assigned to two groups: those without heparin bridging (the control group) and those with temporary discontinuation of antithrombotic agents with heparin bridging therapy (the bridging group). A 10–12-core TRUS biopsy was performed; the patients were evaluated for bleeding-related complications.

**Results:**

Of 1134 patients, 1109 (1281 biopsies) and 25 (26 biopsies) were assigned to the control and bridging group, respectively. Patient background did not significantly differ between the control and bridging groups, except for age, history of diabetes, cardiovascular diseases, and CHADS2 scores. Compared with the control group, the bridging group showed a significantly higher rate of complication for any complication (35 vs. 8.3%, *P* < 0.001), bleeding-related complications (27 vs. 4.4%), and urinary tract infection (7.7 vs. 1.2%). No thromboembolic event was observed in the present study. Multivariate logistic analysis showed that heparin bridging therapy was a significant risk factor for the incidence of any complication and bleeding-related complications.

**Conclusions:**

Heparin bridging therapy with temporal discontinuation of antithrombotic agents may increase the risk of complications after TRUS biopsy. Further, large-scale studies are required to clarify the safety of heparin bridging therapy.

**Electronic supplementary material:**

The online version of this article (doi:10.1186/s40064-016-3610-6) contains supplementary material, which is available to authorized users.

## Background

An estimated 106,032 transrectal ultrasound-guided prostate biopsies (TRUS biopsies) are performed annually in Japan (Kakehi and Naito 2008). TRUS biopsy is the current gold standard for diagnosing prostate cancer and is one of the most commonly performed procedures. The complications of TRUS biopsy have previously been described (Loeb et al. 2013; Nam et al. 2013; Pinkhasov et al. 2012; Rosario et al. 2012). The most frequent of these, which are usually self-limiting, is hematuria (14.7–81.5%) (Giannarini et al. 2007; Ihezue et al. 2005; Kakehi and Naito 2008). Although the majority of patients have minor hematuria without complications, a few (0.3–6.2%) can develop severe problems (Nam et al. 2013; Pinkhasov et al. 2012; Rosario et al. 2012).

As the world’s population ages rapidly, a relatively high proportion of patients are on prescribed medication for comorbidities; these medications include long-term antithrombotic therapy using low-dose aspirin, clopidogrel/ticlopidine, and warfarin. Several prospective studies support the safety of continuation of low-dose aspirin in TRUS biopsy (Giannarini et al. 2007; Maan et al. 2003). Conversely, no recommendation exists regarding the management of non-aspirin antithrombotic agents (warfarin or clopidogrel/ticlopidine) before a TRUS biopsy. Clinicians have three options for management, depending on the risk of thrombosis. First, antithrombotic agents can be continued during procedures associated with low rates of bleeding complications. Second, they can be discontinued for several days prior to the procedure and restarted thereafter. Third, they can be discontinued with bridging anticoagulation using heparin (heparin bridging therapy), administered during the sub-therapeutic window, in patients at high risk of thromboembolic events (Douketis et al. 2008; El-Hakim and Moussa 2010). However, there are few prospective studies regarding the relationship between TRUS biopsy and continuation of antithrombotic agents. Furthermore, the safety of short-term discontinuation of antithrombotic agents with heparin bridging therapy in TRUS biopsy has not been well documented. Therefore, we aimed to assess the safety of heparin bridging therapy for TRUS biopsy in patients requiring temporary discontinuation of antithrombotic agents in the present study.

## Methods

### Patient selection and evaluation

From January 2005 to November 2015, we performed 1307 TRUS-biopsies on 1134 patients in our hospital. We retrospectively investigated the relationship between heparin bridging therapy and the incidence of complications after TRUS biopsy and evaluated the prevalence of these complications in patients not receiving heparin bridging therapy. Patients were assigned to two groups: those without heparin bridging (the control group, n = 1109; 1281 biopsies) and those with temporary discontinuation of antithrombotic agents with heparin bridging therapy (the bridging group, n = 25; 26 biopsies). The control group included patients not administered any antithrombotic agents or those with temporary discontinuation of antithrombotic agents. Clinical indication of heparin bridging therapy was assessed by the physician who had medicated the patient with antithrombotic agents, according to the individual risks of thromboembolism. A 10- to 12-core TRUS biopsy was performed, and patients were surveyed to evaluate any complications and bleeding-related events. The incidence of complications and grades for each group was assessed by reference of medical charts, according to the Common Terminology Criteria for Adverse Events (version 4.0). Multivariate logistic regression analysis was performed to assess the risk factors for the incidence of complications (of any grade), including age, heparin bridging, presence of comorbidities (diabetes mellitus and hypertension), number of biopsy cores, and the CHADS2 score (congestive heart failure: 1 point; hypertension: 1 point; age 75 or older: 1 point; diabetes mellitus: 1 point; prior stroke or transient ischemic attack or thromboembolism: 2 points). The CHADS2 score is a clinical prediction rule for estimating the risk of stroke in patients with non-rheumatic atrial fibrillation that is a common and serious heart arrhythmia associated with thromboembolic stroke (Gage et al. 2001). To evaluate the accuracy and reliability of the CHADS2 score in clinical practice, we compared the international normalized ratio of prothrombin time (PT-INR) and CHADS2 score between heparin bridging and non-bridging heparin-administered patients.

### Statistical analysis

Statistical analyses of the clinical data were performed using SPSS version 22.0 (SPSS Inc., Chicago, IL, USA) and GraphPad Prism 5.03 (GraphPad Software, San Diego, CA, USA). Categorical variables were reported as percentages and compared using the Fisher’s exact test. Quantitative data were expressed as medians with quartiles 1 and 3 (Q1 and Q3). Differences between the groups were statistically compared using the Student’s *t* test for normal distribution or the Mann–Whitney *U*-test for non-parametric distribution. *P* values <0.05 were considered statistically significant. Risk factors for any complications and bleeding-related events were identified using univariate and logistic regression multivariate analyses. Odds ratios (ORs) with 95% confidence intervals were calculated after concurrently controlling for potential confounders. Variables included in the models were age (>70 years), history of type 2 diabetes (positive), history of hypertension (positive), prostate-specific antigen (>7.8 ng/mL), prostate cancer (positive), the number of biopsy cores (>10), use of antithrombotic agents (positive), the number of antithrombotic agents (2 or more), use of warfarin (positive), heparin bridging (positive), and the CHADS2 score (2 or higher). The median was used as a cut-off value in quantitative data.

### Ethics statement

This study was performed in accordance with the ethical standards of the Declaration of Helsinki and approved by the ethics review board of Hirosaki University School of Medicine (Authorization Number: 2013-315). For this type of retrospective study, formal consent was not required.

## Results

Of 1134 patients, 1109 (1281 biopsies) and 25 (26 biopsies) were assigned to the control and bridging groups, respectively. The control group was consisted of the patients without any antithrombotic agents (1013 biopsies) and those with temporary discontinuation of antithrombotic agents (268 biopsies). The rates of any complications and bleeding-related complications in without any antithrombotic agents and those with temporary discontinuation of antithrombotic agents were 7.7 and 10% (*P* = 0.314), 4.5 and 3.7% (*P* = 0.737), respectively. Because no significant differences were observed in complication rates between the groups, we integrated those patients in control group.

In the bridging group, the number of patients with oral antithrombotic agents were as follows; warfarin 13, low-dose aspirin 3, clopidogrel 1, dabigatran 1, warfarin + low-dose aspirin 3, low-dose aspirin + ticlopidine 2, warfarin + low-dose aspirin + ticlopidine 2, and warfarin + low-dose aspirin + cilostazol + sarpogrelate 1.

There were no significant differences in patient backgrounds between the groups, except for age, history of diabetes, use of antithrombotic argents, cardiovascular diseases, and CHADS2 scores (Table 1). Compared with the control group, the patients in the heparin bridging group were significantly older (70 vs. 74 years), had a higher prevalence of diabetes (11 vs. 27%), and a higher CHADS2 score (0 vs. 2 points). The total number of complications and the bleeding-related events were 106 (8.3%) and 57 (4.4%) cases in the control group and 9 (35%) and 7 (27%) cases in the bridging group, respectively. The incidence of complications in the bridging group was significantly higher than that in the control group. However, no significant difference was observed for severe (grade 3 or higher) complications. No thromboembolism-associated complication was observed in the present study (Table 1). Multivariate logistic analysis showed that diabetes, numbers of biopsy cores >10, and heparin bridging were selected as risk factors for the incidence of any complications (Table 2). In bleeding-related events, age >70 and heparin bridging were significant risk factors. Use of non-aspirin antithrombotic agents was not statistically significant for bleeding-related complications (Table 2).

**Table 1:** Patient background, and incidents and rates of complications

	All	Control	Bridging	*P* value
Biopsy, n	1307	1281	26	
Patients, n	1134	1109	25	
Age (years)	70 (65, 74)	70 (65, 70)	74 (71, 76)	<0.001
PSA (ng/mL)	7.8 (5.4, 13.5)	7.8 (5.4, 13.5)	10.5 (6.8, 14.6)	0.099
Diabetes, n	131 (13%)	124 (11%)	7 (27%)	0.011
Hypertension, n	423 (32%)	411 (37%)	12 (46%)	0.141
Frequencies of biopsy	1 (1, 2)	1 (1, 2)	1 (1, 1)	0.970
Number of cores	10 (10, 12)	10 (10, 12)	10 (10, 10)	0.523
Prostate cancer, n	607 (46%)	594 (46%)	13 (50%)	0.843
Use of any antithrombotic agents (any), n	294 (23%)	268 (21%)	26 (100%)	<0.001
Low dose aspirin		191 (15%)	11 (42%)	
Warfarin		52 (4.1%)	19 (73%)	
Clopidogel/ticlodipine		37 (2.9%)	7 (27%)	
NOAC		3 (0.2%)	1 (3.8%)	
Reason for antithrombotic agents use, n				
Atrial fibrillation (Af)	157 (12%)	139 (11%)	18 (69%)	
Myocardial infarction (MI)	52 (4.0%)	46 (3.6%)	6 (23%)	
Others	86 (6.6%)	84 (6.6%)	2 (8%)	
CHADS2 score	0 (0, 0)	0 (0, 1)	2 (0, 2)	<0.001
Complications				
All events, n	118 (9.0%)	106 (8.3%)	9 (35%)	<0.001
Grade 1 or 2	106 (8.1%)	95 (7.4%)	8 (31%)	
Grade 3	12 (0.9%)	11 (0.9%)	1 (3.8%)	
Bleeding related events, n	64 (4.9%)	57 (4.4%)	7 (27%)	<0.001
Grade 1 or 2	57 (4.4%)	51 (4.0%)	6 (23%)	
Grade 3	7 (0.5%)	6 (0.5%)	1 (3.8%)	
Urinary tract infection, n	17 (1.3%)	15 (1.2%)	2 (7.7%)	0.043
Others, n	34 (2.6%)	34 (2.7%)	0 (0%)	1.000
Thrombotic events, n	0 (0%)	0 (0%)	0 (0%)	

Median and interquartile range (Q1, Q3) was used for consecutive variables

*PT-INR* prothrombin time-international normalized ratio, *NOAC* novel oral anticoagulant

**Table 2:** Uni- and multivariate logistic analysis of risk factors for complications

	Risk factors	Any complications			Bleeding-related events		
		*P* value	HR	95% CI	*P* value	HR	95% CI
*Univariate*							
Age	>70 years	0.023	1.57	1.06–2.32	0.004	2.14	1.27–3.61
Diabetes	Positive	0.004	2.09	1.26–3.44	0.052	1.91	0.99–3.67
Hypertension	Positive	0.883	1.03	0.69–1.53	0.802	0.94	0.55–1.58
PSA	>7.8 ng/mL	0.641	0.91	0.62–1.34	0.454	0.82	0.50–1.37
Prostate cancer	Positive	0.641	0.92	0.63–1.33	0.859	0.96	0.59–1.55
Number of biopsy cores	>10 cores	0.063	1.48	0.98–2.23	0.521	1.20	0.69–2.08
Use of oral antithrombotic agents	Positive	0.025	1.62	1.06–2.47	0.425	1.26	0.71–2.23
Number of oral antithrombotic agents	2 or more	0.043	1.38	1.01–1.90	0.281	1.26	0.83–1.92
Use of warfarin	Positive	0.679	1.19	0.53–2.65	0.745	1.19	0.42–3.37
Heparin-bridging	Positive	0.000	5.99	2.61–13.8	0.000	7.91	3.20–19.6
CHADS2 score	2 or higher	0.005	2.30	1.29–4.08	0.003	2.83	1.42–5.61
*Multivariate*							
Age	>70 years	0.108	1.39	0.93–2.09	0.015	1.96	1.14–3.36
Diabetes	Positive	0.023	1.99	1.10–3.59	0.243	1.60	0.73–3.52
Number of biopsy cores	>10 cores	0.025	1.62	1.06–2.47	0.371	1.29	0.74–2.27
Use of oral antithrombotic agents	Positive	0.473	1.19	0.74–1.92	0.280	0.68	0.33–1.38
Heparin-bridging	Positive	0.002	4.78	1.77–13.0	0.001	7.52	2.29–24.8
CHADS2 score	2 or higher	0.912	0.96	0.44–2.06	0.655	1.25	0.47–3.31

Our study included 68 patients who were taking warfarin before TRUS biopsy. Of these, 19 and 49 patients were assigned to warfarin discontinuation with and without heparin bridging (discontinuation alone) groups by the primary physician, respectively. We compared the PT-INR and CHADS2 scores between these two groups. Our data showed that PT-INR was significantly higher in heparin bridging patients. However, CHADS2 score was not significantly different between the groups (Table 3).

**Table 3:** Background of patient who are taking warfarin

	Discontinuation of warfarin		*P* value
	Without bridging	With bridging	
Biopsy, n	49	19	
Patients, n	46	18	
Age (years)	72 (68, 76)	73 (71, 76)	0.406
PSA (ng/mL)	8.8 (5.4, 13.6)	10.5 (7.0, 14.9)	0.333
Diabetes, n	5 (10%)	5 (26%)	0.128
Hypertension, n	23 (47%)	7 (37%)	0.588
Concurrent antithrombotic agents			1.000
Warfarin alone, n	34 (69%)	13 (68%)	
Warfarin + others, n	15 (31%)	6 (32%)	
PT-INR (at a target level)	1.55 (1.19, 1.92)	1.89 (1.67, 2.32)	0.026
CHAD2 score	1 (1, 3)	1 (0, 3)	0.701

Median and interquartile range (Q1, Q3) was used for consecutive variables. PT-INR: prothrombin time-international normalized ratio

To assess the safety of heparin bridging therapy, we compared patients with temporary discontinuation of antithrombotic agents (n = 268) and the bridging group (n = 26). Although there were age differences between the groups, the rates of any complications and bleeding-related complications in those with discontinuation and the bridging group are 9.7 and 35% (*P* = 0.001), 3.7 and 27% (*P* = 0.001), respectively (Table 4).

**Table 4:** Comparison between patients with temporary discontinuation of antithrombotic agents and the bridging group

	Temporary discontinuation of antithrombotic agents	Bridging	*P* value
Biopsy, n	268	26	
Patients, n	235	25	
Age (years)	72 (68, 75)	74 (71, 76)	0.016
PSA (ng/mL)	7.6 (5.3, 13.4)	10.5 (6.8, 14.6)	0.329
Diabetes, n	41 (15%)	7 (27%)	0.160
Hypertension, n	132 (49%)	12 (46%)	0.839
Frequencies of biopsy	1 (1, 1)	1 (1, 1)	0.124
Number of cores	10 (10, 10)	10 (10, 10)	0.526
Prostate cancer, n	131(49%)	13 (50%)	0.913
Use of any antithrombotic agents (any), n	268 (100%)	26 (100%)	
Low dose aspirin	191 (15%)	11 (42%)	
Warfarin	52 (4.1%)	19 (73%)	
Clopidogel/ticlodipine	37 (2.9%)	7 (27%)	
NOAC	3 (0.2%)	1 (3.8%)	
Reason for antithrombotic agents use, n			<0.001
Atrial fibrillation (Af)	139 (52%)	18 (69%)	
Myocardial Infarction (MI)	46 (17%)	6 (23%)	
Others	84 (31%)	2 (8%)	
CHADS2 score	0 (0, 1)	2 (0, 2)	<0.001
*Complications*			
All events, n	26 (9.7%)	9 (35%)	0.001
Grade 1 or 2	22 (8.2%)	8 (31%)	
Grade 3	4 (1.5%)	1 (3.8%)	
Bleeding related events, n	10 (3.7%)	7 (27%)	<0.001
Grade 1 or 2	8 (2.9%)	6 (23%)	
Grade 3	2 (0.7%)	1 (3.8%)	
Urinary tract infection, n	4 (1.5%)	2 (7.7%)	0.002
Others, n	12 (4.5%)	0 (0%)	1.000
Thrombotic events, n	0 (0%)	0 (0%)	

Median and interquartile range (Q1, Q3) was used for consecutive variables

*NOAC* novel oral anticoagulant

## Discussion

The potential risk of severe hemorrhage requiring hospitalization was reportedly 1/500 in men undergoing TRUS biopsy without warfarin administration, with a theoretical fivefold increase in men administered warfarin as an anticoagulant (Ramachandran et al. 2005). When a patient is considered to be at a low or intermediate risk for thromboembolism, most clinicians advocate discontinuation of antithrombotic therapy prior to TRUS biopsy and recommend continuation only after any bleeding has stopped (Cochlin 2005). The risk of thrombotic events associated with stopping warfarin is reportedly approximately 1% (Wahl 1998; Yasaka et al. 2001). Several observational studies have suggested that simply interrupting warfarin therapy for <7 days is associated with a very low rate of thromboembolism and severe bleeding complications (Garcia et al. 2008; Wysokinski et al. 2008). A survey among urologists and radiologists found that 84% of urologists stopped warfarin 4 days before TRUS biopsy, and 95% of radiologists stopped it 5 days before TRUS biopsy (Connor and Wingate 1999). A PT-INR of less than 1.5 is accepted for most elective procedures (Kearon and Hirsh 1997).

When patients have a high risk of thromboembolism, either continuation of antithrombotic agents or discontinuation of antithrombotic agents with heparin bridging therapy is required. As a definitive recommendation regarding the management of antithrombotic agents before TRUS biopsy is yet to be established, the benefits and adverse effects of heparin bridging therapy are debatable. Clinicians have traditionally discontinued antithrombotic agents and started heparin bridging therapy, depending on the risk of thrombotic events (Douketis et al. 2008; El-Hakim and Moussa 2010). The guidelines for the management of antithrombotic agents for endoscopic procedures suggest heparin bridging therapy for higher-risk procedures (e.g., polypectomy) in high-risk patients (e.g., those with atrial fibrillation with mechanical valves, valvular heart disease, and/or a history of cerebrovascular accidents, and those who experience transient ischemic attacks) (Murasaki 2011). A recent prospective study suggested that heparin bridging therapy increased the risk of bleeding complications from interruption alone (0.8%) to bridging (13%) in colonoscopy and oral and ophthalmic surgeries (Garcia et al. 2008). However, there is little reliable evidence for the benefits and harm associated with heparin bridging therapy in TRUS biopsy. To our knowledge, this is the first report to describe the risk of heparin bridging therapy for TRUS biopsy in patients requiring temporary discontinuation of antithrombotic agents. Our data suggested that discontinuation of antithrombotic agents with heparin bridging therapy increased the incidence of complications after TRUS biopsy. Furthermore, there was an increase in bleeding-related complications and urinary tract infections. Although the majority of complication grades were low (1 or 2), the incidence rate of complications was significantly higher in heparin bridging patients than in control patients. Similarly to a previous study (Garcia et al. 2008), our results suggested the possibility that heparin bridging therapy may cause more harm than good. From a different perspective, this is reasonable because patients who are indicated for heparin bridging therapy have a number of severe comorbidities. In fact, our patients treated with heparin bridging therapy had a significantly higher prevalence of diabetes and cardiovascular diseases. Therefore, it is possible that those patients who were recommended heparin bridging therapy have a high risk for complications, and careful management of such complications is required in these patients.

In addition, the patients with temporary discontinuation of antithrombotic agents should be compared to the bridging group to assess the safety of heparin bridging therapy. The rates of any complications and bleeding-related complications in those with discontinuation and the bridging group were 9.7 and 35% (*P* = 0.001), 3.7 and 27% (*P* = 0.001), respectively. This result implies that the heparin bridging itself, in spite of the past use of antithrombotic agents, affects the complication rate. Furthermore, the patient background of these two groups is compared to consider the influence of heparin bridging. The comorbidity rates of diabetes and hypertension in those with discontinuation and the bridging group were 15 and 27% (*P* = 0.160), and 49 and 46% (*P* = 0.839), respectively. This similarity of background also supports the significance of heparin bridging for complication rates.

We observed no thrombotic event in this cohort. Our results may be biased by the limitations of sample size and/or the ethnic differences. The risk of thrombotic disease is different between Japanese and Caucasians (Murasaki 2011). Therefore, we could not establish the benefits or adverse effects of heparin bridging therapy in this present study.

To balance the potential benefits and adverse effects, continuation of antithrombotic agents is another option for reducing thrombotic events. Several studies have suggested the safety of continuation of warfarin amid TRUS- biopsy. In the studies of Ihezue et al. (2005) and Chowdhury et al. (2012), no significant differences were observed in the severity of bleeding between the patients taking warfarin (36.7 and 27.9%, respectively) and the controls (60.2 and 37.0%, respectively). These results suggest that discontinuation of warfarin before TRUS biopsy is unnecessary. However, the limitations of these studies include the non-randomized design, potentially life-threatening hemorrhagic complications that may have been avoided only due to the sample size and limited information regarding the proper control of PT-INR. Another alternative is a switch of novel oral anticoagulant (NOAC). It may decrease the risk of bleeding and thromboembolism (Douketis et al. 2014). However, there is limited evidence for invasive procedures with interruption of NOAC administration. Further studies are needed to evaluate the benefits and adverse effects of discontinuation alone, continuation, or discontinuation with administration of heparin bridging therapy in TRUS biopsy.

To carefully assess the benefits and adverse effects, risk classification is necessary. One of the major risk classifications is the CHADS2 score (Gage et al. 2004). It is used to determine whether or not antithrombotic treatment is required. The CHADS2 score is simple and its applicability has been validated by many studies. Based on the CHADS2 score, this decision pertaining to the treatment modality should be discussed with the patient and the primary physician managing the antithrombotic agents. To assess the accuracy and reliability of CHADS2 scores in clinical practice, we selected 68 patients who were taking warfarin and compared the PT-INR and CHADS2 scores between patients with and without heparin bridging therapy. Our data showed that the target level of PT-INR was significantly higher in patients with heparin bridging therapy. However, no relationship was observed between the CHADS2 score and a recommendation for heparin bridging therapy. This finding suggests the difficulty in truly defining risk stratification and decision making, and highlights the urgent need for large-scale randomized clinical trials to evaluate the management of continuation of antithrombotic agents or bridging therapy in patients undergoing TRUS biopsy.

The present study has several limitations, including small sample size, its retrospective nature, and the indication of heparin bridging therapy. Among these, the retrospective design may result in remarkable biases in this study. For instance, propensity score matching was not applied because of the small sample size. On the other hand, because the judgement regarding antithrombotic therapy possibly causes fatal complications such as intracranial hemorrhage and pulmonary embolism, the prospective study needs to be designed very carefully in the future. Although our study suggested that heparin bridging therapy may increase the risk of bleeding complications, safety of this therapy remains inconclusive. Despite these limitations, this study is the first to report the safety of heparin bridging therapy for TRUS biopsy in patients requiring temporary discontinuation of antithrombotic agents (Additional file 1).

## Conclusion

In conclusion, bridging of temporal discontinuation of antithrombotic agents with heparin may increase the risk of complications. However, the limited number of patients with heparin bridging therapy obfuscates a clear interpretation of the results. Further large-scale studies are necessary to clarify the benefits and adverse effects of heparin bridging therapy.

## Additional file

**Additional file 1.** Patient background and adverse events of all 1307 prostate biopsies.

## Abbreviations

TRUS biopsy: transrectal ultrasound-guided prostate biopsy
ASA: acetylsalicylic acid
AEs: adverse events
PT-INR: prothrombin time-international normalized ratio
NOAC: non-vitamin K antagonist oral anticoagulation
